# Preexisting immune‐mediated inflammatory disease is associated with improved survival and increased toxicity in melanoma patients who receive immune checkpoint inhibitors

**DOI:** 10.1002/cam4.4239

**Published:** 2021-10-14

**Authors:** Nicholas Gulati, Arda Celen, Paul Johannet, Janice M. Mehnert, Jeffrey Weber, Michelle Krogsgaard, Iman Osman, Judy Zhong

**Affiliations:** ^1^ The Ronald O. Perelman Department of Dermatology New York University Grossman School of Medicine New York NY USA; ^2^ Department of Medicine New York University Grossman School of Medicine New York NY USA; ^3^ Department of Pathology New York University Grossman School of Medicine New York NY USA; ^4^ Department of Population Health New York University Grossman School of Medicine New York NY USA

**Keywords:** cancer management, clinical cancer research, immunology, melanoma

## Abstract

**Background:**

Immune‐related adverse events (irAEs) are common, clinically significant autoinflammatory toxicities observed with immune checkpoint inhibitors (ICI). Preexisting immune‐mediated inflammatory disease (pre‐IMID) is considered a relative contraindication to ICI due to the risk of inciting flares. Improved understanding of the risks and benefits of treating pre‐IMID patients with ICI is needed.

**Methods:**

We studied melanoma patients treated with ICI and enrolled in a prospective clinicopathological database. We compiled a list of 23 immune‐mediated inflammatory diseases and evaluated their presence prior to ICI. We tested the associations between pre‐IMID and progression‐free survival (PFS), overall survival (OS), and irAEs.

**Results:**

In total, 483 melanoma patients were included in the study; 74 had pre‐IMID and 409 did not. In patients receiving ICI as a standard of care (SoC), pre‐IMID was significantly associated with irAEs (*p* = 0.04) as well as improved PFS (*p* = 0.024) and OS (*p* = 0.007). There was no significant association between pre‐IMID and irAEs (*p* = 0.54), PFS (*p* = 0.197), or OS (*p* = 0.746) in patients treated through a clinical trial. Pre‐IMID was significantly associated with improved OS in females (*p* = 0.012), but not in males (*p* = 0.35).

**Conclusions:**

The dichotomy of the impact of pre‐IMID on survival and irAEs in SoC versus clinical trial patients may reflect the inherit selection bias in patients accrued in clinical trials. Future mechanistic work is required to better understand the differences in outcomes between female and male pre‐IMID patients. Our data challenge the notion that clinicians should avoid ICI in pre‐IMID patients, although close monitoring and prospective clinical trials evaluating ICI in this population are warranted.

## Graphical abstract

Melanoma patients with preexisting immune‐mediated inflammatory diseases have improved survival and increased toxicity upon treatment with immune checkpoint inhibitors. This challenges the notion that clinicians should avoid these therapies in patients with preexisting immune‐mediated inflammatory diseases, but close monitoring is warranted.

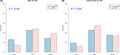

## 1 BACKGROUND

Immune checkpoint inhibitors (ICI) improve progression‐free survival (PFS) and overall survival (OS) for a subset of patients with advanced melanoma. However, many individuals develop immune‐related adverse events (irAEs), which are autoinflammatory toxicities that can mimic the clinical presentation of conventional immune‐mediated inflammatory diseases.^1^ Preexisting immune‐mediated inflammatory disease (pre‐IMID) is considered a relative contraindication to treatment with ICI due to the concerns of inciting an immune‐mediated flare or increasing the risk of developing additional irAEs. Therefore, patients with pre‐IMID have generally been excluded from clinical trials of ICI. This creates a large unmet clinical need as pre‐IMID patients are more likely to have a malignancy due to chronic inflammation and treatment‐related immunosuppressive effects.^2^, ^3^ In fact, patients with newly diagnosed melanoma have a 28.3% prevalence rate of pre‐IMID, a rate that has been increasing with time.^4^

Given the large number of pre‐IMID patients who would potentially benefit from ICI treatment, pre‐IMID is not considered to be an absolute contraindication to the use of ICI in current standard of care (SoC) clinical practice. Nevertheless, many oncologists remain justifiably wary of prescribing ICI to pre‐IMID patients since the true risk–benefit ratio is unclear.^5^

We, therefore, aimed to determine whether pre‐IMID status impacts the likelihood of responding to immunotherapy and/or developing toxicity. To do this, we examined a cohort of melanoma patients who received ICI either through a clinical trial or as per SoC clinical practice. We considered a wide range of immune‐mediated inflammatory diseases with diverse pathogeneses in our analysis, and tested the impact of pre‐IMID on both PFS and OS, as well as on irAEs, in melanoma patients treated with ICI.

## 2 METHODS

### 2.1 Patient characteristics

The cohort includes patients with melanoma who received care at New York University Langone Health between 2003 and 2021. All patients provided written informed consent to be enrolled in a database with prospective follow‐up (institutional review board #10362), and the research conformed to the standards of the Declaration of Helsinki. Patients were classified as having pre‐IMID or no pre‐IMID depending on whether or not they had an immune‐mediated inflammatory disease prior to the start of ICI. If a patient had more than one line of ICI treatment, they were classified as having pre‐IMID only if the diagnosis was present before their first line of ICI. A treatment line is defined as a time period (which can be of various lengths depending on the treating oncologist's prescribing practice) when a patient received any type of ICI treatment. If the patient then received another ICI treatment (whether the same or different agent) after a period off of ICI treatment, that is considered a new treatment line. The list of immune‐mediated inflammatory diseases that we examined was derived from a prior publication,^6^ and included: asthma, celiac disease, chronic inflammatory demyelinating polyneuropathy, dermatomyositis, eczema, erythema nodosum, Graves’ disease, Guillain‐Barré syndrome, idiopathic thrombocytopenia, inflammatory bowel disease (either Crohn's disease or ulcerative colitis), multiple sclerosis, myasthenia gravis, polymyalgia rheumatica, psoriasis, psoriatic arthritis, rheumatoid arthritis, sarcoidosis, scleroderma, Sjogren's syndrome, systemic lupus erythematosus, transverse myelitis, and vasculitis.

### 2.2 Statistical analysis

Baseline characteristics between patients with and without pre‐IMID were compared using the Chi‐square test. Kaplan–Meier curves were generated and compared by the log‐rank test to estimate OS and PFS for each group. Multivariable cox proportional hazard models were generated to further analyze the associations between pre‐IMID and PFS, OS, and irAE development. The multivariable analysis was adjusted for sex, age, stage, Eastern Cooperative Oncology Group (ECOG) status, and treatment line (first line versus second or third line). Multiple treatment lines were clustered by patient IDs in the multivariable cox proportional hazard models.

## 3 RESULTS

### 3.1 Patients with pre‐IMID were significantly more likely to experience toxicity than patients without pre‐IMID

Table 1 shows the baseline characteristics of the cohort. Out of the 483 patients included in this study, there were 74 (15.3%) who had 83 different pre‐IMID (seven patients had two diseases and one had three diseases). The most common conditions observed in the patients were asthma (*n* = 42), inflammatory bowel disease (*n* = 10), psoriasis (*n* = 9), rheumatoid arthritis (*n* = 8), and eczema (*n* = 6) (Table 2). Table 3 categorizes the 716 treatment lines received by patients with (*n* = 122) and without (*n* = 594) pre‐IMID. The cohorts were generally well balanced in terms of their baseline demographics and clinical characteristics. There were no significant differences between the two groups with respect to age, sex, ECOG status, stage, treatment indication (metastatic/recurrence versus adjuvant/neoadjuvant), number of metastatic sites, number of treatment lines, line of ICI treatment (first line versus second or third line), and being part of SoC versus clinical trial. Patients in the no pre‐IMID group were significantly more likely to be non‐Hispanic White compared to the pre‐IMID group. Also, patients in the no pre‐IMID group were significantly more likely to have received anti‐cytotoxic T‐lymphocyte‐associated antigen 4 (CTLA‐4) monotherapy as opposed to anti‐programmed death 1 (PD‐1) monotherapy or combination anti‐CTLA‐4 plus anti‐PD‐1 therapy. Patients in the pre‐IMID group were significantly more likely to experience mild or severe toxicity compared to the no pre‐IMID group (*p* = 0.038).

**TABLE 1:** Patient characteristics (*n* = 483)

Characteristic	*n* (%)
Age (mean[SD])	62.83 (15.21)
Sex	
Male	295 (61.1)
Female	188 (38.9)
Ethnicity	
Non‐Hispanic White	431 (91.1)
Non‐Hispanic Black	12 (2.5)
Non‐Hispanic Asian or Pacific Islander	8 (1.7)
Hispanic	18 (3.8)
Non‐Hispanic American Indian/Alaska Native	3 (0.6)
Other	1 (0.2)

Abbreviation: SD, standard deviation.

**TABLE 2:** List of preexisting immune‐mediated inflammatory diseases present in our cohort of melanoma patients treated with ICI, categorized by sex

Immune‐mediated inflammatory disease	Number of patients	Females	Males
Asthma	42	22	20
Inflammatory bowel disease (Crohn's disease plus ulcerative colitis)	10	4	6
Psoriasis (including Psoriatic arthritis)	9	2	7
Rheumatoid arthritis	8	5	3
Eczema	6	2	4
Polymyalgia rheumatica	3	1	2
Celiac disease	1	1	0
Sarcoidosis	1	1	0
Scleroderma	1	0	1
Lupus	1	1	0
Graves’ disease	1	1	0

**TABLE 3:** Characteristics of treatment lines with (pre‐IMID) and without (no pre‐IMID) preexisting immune‐mediated inflammatory diseases

Characteristic	Overall	No pre‐IMID	Pre‐IMID	*p*
*n*	716	594	122	
Age (mean [SD])	63.29 (14.84)	63.24 (14.91)	63.54 (14.54)	0.841
Sex (%)				
Female	295 (41.2)	238 (40.1)	57 (46.7)	0.208
Male	421 (58.8)	356 (59.9)	65 (53.3)	
Race (%)				
Hispanic	27 (3.9)	18 (3.1)	9 (7.6)	0.031
Non‐Hispanic American Indian/Alaska Native	5 (0.7)	2 (0.3)	3 (2.5)	
Non‐Hispanic Asian or Pacific Islander	14 (2.0)	12 (2.1)	2 (1.7)	
Non‐Hispanic Black	19 (2.7)	16 (2.8)	3 (2.5)	
Non‐Hispanic White	633 (90.6)	531 (91.6)	102 (85.7)	
Other	1 (0.1)	1 (0.2)	0 (0.0)	
ECOG status (mean [SD])	0.34 (0.58)	0.35 (0.58)	0.33 (0.57)	0.796
Stage (%)				
II	15 (2.1)	13 (2.2)	2 (1.6)	0.878
III	166 (23.2)	139 (23.4)	27 (22.1)	
IV	535 (74.7)	442 (74.4)	93 (76.2)	
Treatment Indication (%)				
Adjuvant/neoadjuvant	125 (17.5)	105 (17.7)	20 (16.4)	0.834
Metastatic/recurrence	591 (82.5)	489 (82.3)	102 (83.6)	
Number of metastatic sites (mean [SD])	2.02 (1.60)	2.04 (1.62)	1.93 (1.48)	0.459
Number of treatment lines (mean [SD])	1.88 (1.08)	1.85 (1.08)	2.03 (1.06)	0.084
Treatment line number (%)				
Second or third	237 (33.1)	188 (31.6)	49 (40.2)	0.086
First	479 (66.9)	406 (68.4)	73 (59.8)	
ICI type (%)				
Anti‐CTLA−4	222 (31.0)	202 (34.0)	20 (16.4)	<0.001
Anti‐CTLA−4 + Anti‐PD−1	213 (29.7)	165 (27.8)	48 (39.3)	
Anti‐PD−1	281 (39.2)	227 (38.2)	54 (44.3)	
SoC versus trial (%)				
SoC	533 (74.4)	445 (74.9)	88 (72.1)	0.597
Trial	183 (25.6)	149 (25.1)	34 (27.9)	
Overall toxicity (%)				
None	158 (22.5)	142 (24.3)	16 (13.7)	0.038
Mild	324 (46.2)	266 (45.5)	58 (49.6)	
Severe	220 (31.3)	177 (30.3)	43 (36.8)	

Abbreviations: CTLA‐4, cytotoxic T‐lymphocyte‐associated antigen 4; ECOG, Eastern Cooperative Oncology Group; ICI, immune checkpoint inhibitors; PD‐1, programmed death 1; pre‐IMID, preexisting immune‐mediated inflammatory disease; SD, standard deviation; SoC, standard of care.

### 3.2 Patients with pre‐IMID who received ICI as part of SoC had significantly improved survival and increased toxicity, but those who received ICI in a clinical trial did not

**FIGURE 1:**
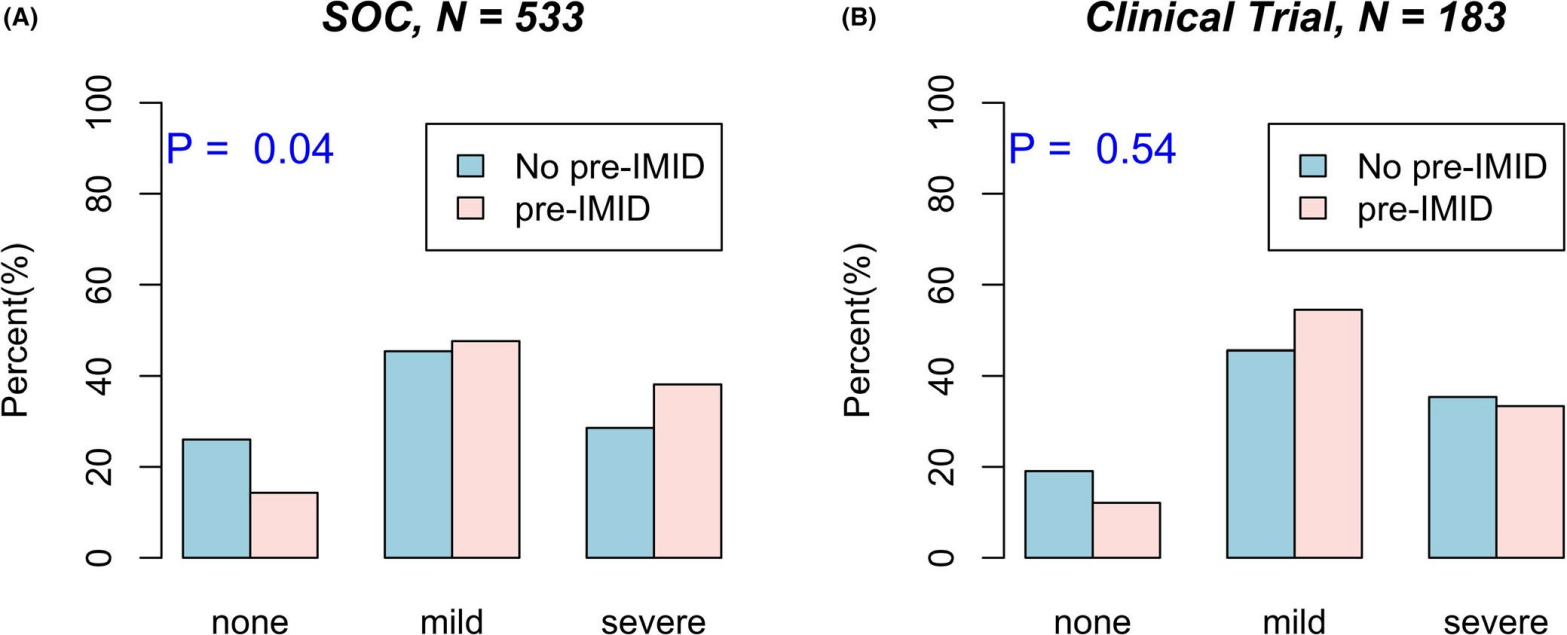
Pre‐IMID is significantly associated with overall toxicity in melanoma patients receiving ICI as part of SoC but not through a clinical trial. Rates of no, mild, and severe overall toxicity in melanoma patients receiving ICI through (A) SoC (*N* = 533) and (B) clinical trial (*N* = 183) by pre‐IMID grouping

Out of 716 treatment lines, there were 533 (74.4%) that were administered as a part of SoC, and 183 (25.6%) that were administered through a clinical trial. We found that 88/533 (16.5%) lines in the SoC group were in patients with pre‐IMID. For the clinical trial group, 34/183 (18.6%) treatment lines were in patients with pre‐IMID. Pre‐IMID was associated with a higher probability of experiencing mild (grades 1–2) or severe (grades 3–5) toxicity in the SoC group (*p* = 0.04), but not in the trial group (*p* = 0.54; Figure 1). By multivariable Cox proportional hazard model adjusting for age, sex, stage, ECOG status, and line of ICI treatment (first line versus second or third line), pre‐IMID was significantly associated with PFS (adjusted hazard ratio [aHR] = 0.49 [95% CI: 0.26, 0.91], *p* = 0.024) and OS (aHR = 0.21 [0.07, 0.65], *p* = 0.007) in the SoC group, but not in the trial group (aHR = 1.74 [0.75, 4.03], *p* = 0.197 and aHR = 0.82 [0.25, 2.69], *p* = 0.746, respectively). Table 4 categorizes the 716 treatment lines received by patients as either a part of SoC (*n* = 533) or through a clinical trial (*n* = 183). There were no significant differences between the two groups with respect to sex, race, stage, treatment indication, number of metastatic sites, ICI agent administered, and toxicity. SoC patients were significantly older and had significantly higher ECOG scores than trial patients. Also, SoC patients were significantly more likely to have more than one line of ICI treatment compared to trial patients.

**TABLE 4:** Characteristics of treatment lines as part of standard of care (SoC) and clinical trial

Characteristic	Overall	SoC	Trial	*p*
*n*	716	533	183	
Age (mean [SD])	63.29 (14.84)	64.19 (15.35)	60.65 (12.93)	0.005
Sex (%)				
Female	295 (41.2)	228 (42.8)	67 (36.6)	0.169
Male	421 (58.8)	305 (57.2)	116 (63.4)	
Race (%)				
Hispanic	27 (3.9)	22 (4.2)	5 (2.8)	0.288
Non‐Hispanic American Indian/Alaska Native	5 (0.7)	2 (0.4)	3 (1.7)	
Non‐Hispanic Asian or Pacific Islander	14 (2.0)	12 (2.3)	2 (1.1)	
Non‐Hispanic Black	19 (2.7)	16 (3.1)	3 (1.7)	
Non‐Hispanic White	633 (90.6)	467 (89.8)	166 (92.7)	
Other	1 (0.1)	1 (0.2)	0 (0.0)	
ECOG status (mean [SD])	0.34 (0.58)	0.39 (0.63)	0.21 (0.41)	0.001
Stage (%)				
II	15 (2.1)	10 (1.9)	5 (2.7)	0.712
III	166 (23.2)	126 (23.6)	40 (21.9)	
IV	535 (74.7)	397 (74.5)	138 (75.4)	
Treatment Indication (%)				
Adjuvant/neoadjuvant	125 (17.5)	89 (16.7)	36 (19.7)	0.423
Metastatic/recurrence	591 (82.5)	444 (83.3)	147 (80.3)	
Number of metastatic sites (mean [SD])	2.02 (1.60)	2.03 (1.59)	2.01 (1.63)	0.9
Number of treatment lines (mean [SD])	1.88 (1.08)	1.93 (1.07)	1.74 (1.09)	0.038
Treatment line number (%)				
Second or third	237 (33.1)	194 (36.4)	43 (23.5)	0.002
First	479 (66.9)	339 (63.6)	140 (76.5)	
ICI type (%)				
Anti‐CTLA−4	222 (31.0)	173 (32.5)	49 (26.8)	0.357
Anti‐CTLA−4 + Anti‐PD−1	213 (29.7)	155 (29.1)	58 (31.7)	
Anti‐PD−1	281 (39.2)	205 (38.5)	76 (41.5)	
Overall toxicity (%)				
None	158 (22.5)	126 (24.1)	32 (17.8)	0.174
Mild	324 (46.2)	239 (45.8)	85 (47.2)	
Severe	220 (31.3)	157 (30.1)	63 (35.0)	

Abbreviations: CTLA‐4, cytotoxic T‐lymphocyte‐associated antigen 4; ECOG, Eastern Cooperative Oncology Group; ICI, immune checkpoint inhibitors; PD‐1, programmed death 1; SD, standard deviation; SoC, standard of care.

### 3.3 Females, but not males, with pre‐IMID had significantly improved OS

**FIGURE 2:**
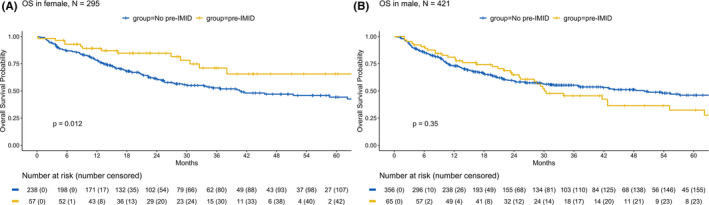
Pre‐IMID is associated with improved OS in female but not in male melanoma patients treated with ICI. OS in (A) female and (B) male melanoma patients by pre‐IMID grouping. All *p*‐values are from the log‐rank tests

When combining both the SoC and trial groups, we found that the presence of pre‐IMID was significantly associated with improved OS in females (*p* = 0.012), but not in males (*p* = 0.35; Figure 2). Similarly, there was a trend toward improved PFS in females with pre‐IMID (*p* = 0.2), but not in males (*p* = 0.95). Neither sex had statistically significant differences in toxicity development in pre‐IMID compared to no pre‐IMID patients (*p* = 0.12 for females, *p* = 0.07 for males).

## 4 DISCUSSION

Our study examines the impact of pre‐IMID on survival and development of irAEs in melanoma patients treated with ICI. Our initial expectation was that patients who received ICI as part of a clinical trial would not have pre‐IMID, due to patients with pre‐IMID traditionally being excluded from clinical trials. However, we surprisingly found that a substantial percentage of treatment lines in both the SoC and trial groups was in patients with pre‐IMID (16.5% and 18.6%, respectively). This prompted us to further examine any potential differences between these two groups. We found statistically significant improvement in survival and increased toxicity in pre‐IMID patients who received ICI only as part of SoC, but not through clinical trials. This emphasizes the importance of examining ICI outcomes in “real world” settings, and of appreciating that clinical trial participants only constitute a select subset of melanoma patients, who may have underlying immune‐mediated inflammatory disease severity that is not fully representative of the population at large. The cancer burden in clinical trial patients may also be lower than the SoC group, as patients with more uncontrolled disease tend to be treated in SoC settings as opposed to clinical trials. In support of this, we found that SoC patients were significantly older, and had significantly higher ECOG scores, than trial patients. Also, SoC patients were significantly more likely to have multiple lines of ICI treatment. This is expected given the fact that the major clinical trials that led to the approval of ICI excluded patients with ECOG scores greater than 1 and those who had received prior treatment for their melanoma. It is possible that the relatively larger number of treatment lines helped lead to the improved survival and increased toxicity we observed for pre‐IMID patients only in the SoC group. One hypothesis is that the “second hit” of ICI treatment that many pre‐IMID SoC patients had was necessary to manifest the improved survival and increased toxicity, due to heighted immune system activation with multiple treatments.

Our findings are consistent with the growing body of evidence that a predisposition to immune‐mediated inflammatory disease may correlate with improved outcomes as well as increased toxicity in patients treated with immune‐modulating medications. Earlier work on melanoma patients treated with interferon alfa‐2b found that the presence of autoantibodies or clinical autoimmune disease is associated with statistically significant improvements in relapse‐free survival and OS.^7^ However, this study focused on autoantibodies or manifestations of autoimmune disease that developed with treatment, and specifically excluded preexisting conditions. Therefore, these patients likely had a predisposition to autoimmunity that became clinically manifest upon interferon alfa‐2b treatment, but did not have overt pre‐IMID as defined in our study. More recent work on patients with non‐small cell lung cancer treated with ICI found that the presence of select preexisting IMID‐related autoantibodies (rheumatoid factor, antinuclear antibody, antithyroglobulin, and antithyroid peroxidase) was associated with significantly improved PFS and increased rates of irAEs.^8^ Further support for the association between autoantibodies and irAE development comes from a study evaluating melanoma patients with high‐throughput protein arrays that identified serum autoantibodies in patients prior to ICI. The results suggest that measurement of pretreatment serum autoantibodies from patients without overt pre‐IMID can predict the development and severity of irAEs.^9^

Interestingly, we found that females with pre‐IMID had significantly improved OS (and a trend toward improved PFS), but males did not. This is despite neither sex having statistically significant differences in toxicity development in pre‐IMID compared to no pre‐IMID patients. It is established that autoimmune disease is more common in women in general, but the reason for this is still under active investigation. There is evidence that male‐predominant autoimmune diseases are characterized by a Th1‐type response, while those in women tend to be more Th2‐ and antibody‐mediated.^10^ Since the presence of autoantibodies is associated with improved OS in cancer patients as noted above, this may help explain the differences we observed between sexes, but future mechanistic work and examination of autoantibodies are required. In our study, there was no significant difference in the specific immune‐mediated inflammatory disease diagnoses rendered between females and males (*p* = 0.354, Table 2). Most conditions, including asthma, were seen in similar numbers of females and males. However, more males than females had psoriasis, which is thought to be more Th1‐ than Th2‐mediated. Future studies with larger sample sizes are needed to see which, if any, specific immune‐mediated inflammatory diseases are overrepresented in females versus males.

The extant literature regarding treatment with ICI in pre‐IMID patients comes mostly from small retrospective case series, which usually focus exclusively on the development of toxicity.^11^ In 2018, a systematic review examined 123 patients from 49 different publications on the subject, and noted that 92/123 (75%) developed exacerbations of pre‐IMID, irAEs, or both.^12^ More recently, a nationwide cohort study in the Netherlands of 4367 patients with melanoma focused on both toxicity and survival. In the 228 patients with select pre‐IMID who received ICI, severe irAE development, response, and survival rates were not significantly different between patients with and without pre‐IMID.^13^ Our findings of improved survival and increased toxicity in pre‐IMID patients are in contrast to this study. This is possibly due to inclusion of a more limited number of autoimmune diseases in the previously published study, which focused on inflammatory bowel disease as well as endocrine and rheumatic conditions. Notably, asthma was not included, and this is not generally thought of as a traditional autoimmune disease, but rather an immune‐mediated inflammatory disease resulting from an allergic‐type reaction to an inhaled substance from the environment.^14^ Asthma was the most frequent immune‐mediated inflammatory disease found in our study, which may account for the disparate results. Similar to the male–female dichotomy noted above, most autoimmune diseases are thought to be Th1‐driven, while asthma is primarily mediated by Th2 cells.^15^ Various serum autoantibodies have been found in asthma patients,^16^ which may be contributing to the improved survival and increased toxicity that we observed. Of note, we found that when comparing organ‐specific irAEs between the pre‐IMID and no pre‐IMID groups, the former group was more likely to experience gastrointestinal, skin, and other irAEs, while the latter group was more likely to experience endocrine and nervous system irAEs. These differences only trended toward statistical significance, but future work correlating pre‐IMID with organ‐specific irAEs is warranted.

There are several limitations to this study. First, there was a relatively small sample size for immune‐mediated inflammatory diseases other than asthma. Second, we had minimal information on the severity of patients’ immune‐mediated inflammatory conditions. Third, although all immune‐mediated inflammatory diseases were determined to be preexisting based on chart review noting presence before ICI treatment, the actual diagnosis date was often unavailable, so some patients may have had a more remote history of disease than others. It is possible that clinical trial patients had less severe or more remote histories of immune‐mediated inflammatory disease than the SoC patients, as more active immune‐mediated inflammatory disease would have likely gotten them excluded from the trial. In turn, this relatively milder immune‐mediated inflammatory disease phenotype in the clinical trial patients may explain the lack of association with survival and toxicity that we observed in this group. One potential confounder to our results is that the no pre‐IMID group was significantly more likely to have received anti‐CTLA‐4 monotherapy as opposed to anti‐PD‐1 monotherapy or combination anti‐CTLA‐4 plus anti‐PD‐1 therapy. Since these different ICI regimens have been shown to have disparate impacts on survival,^1^ the fact that the no pre‐IMID and pre‐IMID groups were not balanced for treatment regimen may be impacting the survival differences that we observed. Given this and all previously published studies are retrospective in nature, there is great need for prospective evaluation of the use of ICI in pre‐IMID patients.

Overall, our data challenge the notion that clinicians should avoid treating pre‐IMID patients with ICI. Multidisciplinary care that includes close collaborations between oncologists and providers who care for patients with organ‐specific immune‐mediated inflammatory diseases such as rheumatologists, pulmonologists, dermatologists, and gastroenterologists is essential for the careful monitoring of pre‐IMID patients receiving ICI. Such collaborations are already in place at many major academic medical centers both in person^17^ and virtually.^18^ Future mechanistic research is needed to understand how to uncouple ICI response from toxicity, and lessons learned from immune‐mediated inflammatory diseases will likely help shed light on the underlying immunology.

## CONFLICT OF INTEREST

The authors declare no relevant conflict of interest.

## ETHICAL APPROVAL STATEMENT

All patients provided written informed consent to be enrolled in a database with prospective follow‐up (institutional review board #10362), and the research conformed to the standards of the Declaration of Helsinki.

## Data Availability

The data that support the findings of this study are available from the corresponding author upon reasonable request.
